# Cardiac output monitoring with pulmonary versus trans-cardiopulmonary thermodilution in left ventricular assist devices: Interchangeable methods?

**DOI:** 10.3389/fphys.2022.889190

**Published:** 2022-09-02

**Authors:** Begoña Quintana-Villamandos, Mónica Barranco, Ignacio Fernández, Manuel Ruiz, Juan Francisco Del Cañizo

**Affiliations:** ^1^ Department of Anesthesiology and Intensive Care, Gregorio Marañón Hospital, Madrid, Spain; ^2^ Department of Pharmacology and Toxicology, Faculty of Medicine, Universidad Complutense, Madrid, Spain; ^3^ Department of Cardiovascular Surgery, Gregorio Marañón Hospital, Madrid, Spain; ^4^ Department of Surgery, Faculty of Medicine, Universidad Complutense, Madrid, Spain

**Keywords:** cardiac output, continuous-flow LVAD, minipig, pulmonary thermodilution, transpulmonary thermodilution

## Abstract

Cardiac output (CO) measurement is mandatory in patients with left ventricular assist devices (LVADs). Thermodilution with pulmonary artery catheter (PAC) remains the clinical gold standard to measure CO in these patients, however it is associated with several complications. Therefore, the agreement between PAC and new, minimally invasive monitoring methods in LVAD needs to be further investigated. The aim of this study was to assess the accuracy and reliability of transpulmonary thermodilution with a PiCCO2 monitor compared with pulmonary artery thermodilution with PAC in a LVAD. Continuous-flow LVADs were implanted in six mini-pigs to assist the left ventricle. We studied two methods of measuring CO—intermittent transpulmonary thermodilution (CO_TPTD_) by PiCCO2 and intermittent pulmonary artery thermodilution by CAP, standard technique (CO_PTD_)—obtained in four consecutive moments of the study: before starting the LVAD (basal moment), and with the LVAD started in normovolemia, hypervolemia (fluid overloading) and hypovolemia (shock hemorrhage). A total of 72 paired measurements were analysed. At the basal moment, CO_TPTD_ and CO_PTD_ were closely correlated (*r* ^2^ = 0.89), with a mean bias of −0.085 ± 0.245 L/min and percentage error of 16%. After 15 min of partial support LVAD, CO_TPTD_ and CO_PTD_ were closely correlated (*r* ^2^ = 0.79), with a mean bias of −0.040 ± 0.417 L/min and percentage error of 26%. After inducing hypervolemia, CO_TPTD_ and CO_PTD_ were closely correlated (*r* ^2^ = 0.78), with a mean bias of −0.093 ± 0.339 L/min and percentage error of 13%. After inducing hypovolemia, CO_TPTD_ and CO_PTD_ were closely correlated (*r* ^2^ = 0.76), with a mean bias of −0.045 ± 0.281 L/min and percentage error of 28%. This study demonstrates a good agreement between transpulmonary thermodilution by PiCCO monitor and pulmonary thermodilution by PAC in the intermittent measurement of CO in a porcine model with a continuous-flow LVAD.

## Introduction

In the last decades, mechanical circulatory support has emerged as an option for patients with heart failure resistant to pharmacotherapy (Tickoo and Bardia, 2020). However, this therapy is associated with mortality, especially during the first 30 days postimplantation (Nepomuceno et al., 2020). Thus, cardiac output (CO) monitoring is essential in patients with left ventricular assist devices (LVADs) (Birati and Rame, 2014; Uriel et al., 2016). Pulmonary artery catheter (PAC) by pulmonary thermodilution is the standard technique for monitoring CO in patients with LVADs (Saxena et al., 2020). However, severe complications have been associated with this invasive form of hemodynamic monitoring in critically ill patients (Bossert et al., 2006).

New and less invasive hemodynamic monitoring techniques have emerged in recent years (Mateu Campos et al., 2012). The PiCCO (pulse index continuous cardiac output) system is currently the only monitor that uses transpulmonary thermodilution to measure CO. It is a minimally invasive form of monitoring (it requires only an arterial and a venous access) that provides information on blood flows and intravascular volumes (Mateu Campos et al., 2012. A PiCCO monitor can measure CO by pulse-contour analysis (continuously) or transpulmonary thermodilution (intermittently) (Halvorsen et al., 2006). Transpulmonary thermodilution is increasingly used for critically ill patients (Teboul et al., 2016). However, it has not yet been validated for hemodynamic monitoring in circulatory support devices.

Thus, in this study, we assess the agreement of the measured values of the PiCCO transpulmonary thermodilution CO (CO_TPTD_) with the pulmonary artery thermodilution CO (CO_PTD_) in a porcine model with a continuous-flow LVAD with normovolemia, hypervolemia (fluid overload), and hypovolemia (bleeding).

## Materials and methods

The study was carried out in the Experimental Medicine and Surgery Unit of the Gregorio Marañón University General Hospital (ES280790000-087). The study was performed in accordance with European Union guidelines on the protection of animals used for experimental and other scientific purposes (Directive 2010/63/EU and Spanish Royal Decree RD 53/2013 BOE) and was approved by the Ethics Committee on animal studies at our institution.

### Anaesthesia and surgical protocol

We use six healthy mini-pigs with a mean weight of 42.8 ± 9.9 kg. The previously described anesthetic protocol was applied (Morillas-Sendín et al., 2015; Quintana-Villamandos et al., 2021). After premedication with 20 mg/kg intramuscular ketamine (Ketolar, Parke-Davis, Madrid, Spain) and 0.04 mg/kg atropine (Atropina Braun, Serra Pamies, Reus, Spain), anesthesia was induced with 2.5 μg/kg intravenous fentanyl (Fentanest, Kern Pharma, Barcelona, Spain) and 4 mg/kg propofol (Diprivan 1%, AstraZeneca, Madrid, Spain). After tracheal intubation, each animal was connected to a volume-controlled ventilator (Dräger SA1, Dräger Medical AG, Lübeck, Germany) with an FIO_2_ of 1, an inspiratory-to-expiratory ratio of 1:2, a tidal volume of 12–15 ml/kg and a respiratory rate adjusted to maintain normocapnia. Anesthesia was maintained with intravenous fentanyl (2.5 μg/kg for 30 min) and propofol in continuous infusion (11–12 mg/kg/h). All animals received an infusion of saline solution (8 ml/kg/h). The PAC (7.5 F Swan-Ganz CCOmbo catheter, Edwards Lifesciences, Irvine, CA, United States) was inserted into the right internal jugular vein for intermittent CO measurement by pulmonary thermodilution and connected to a monitor (Vigilance, Edwards Critical Care Division, Irvine, CA, United States). We used a PiCCO2 set (Pulsion Medical Systems, Munich, Germany) for intermittent CO measurement by transpulmonary thermodilution that consisted of three components: a catheter (5F, 20 cm) inserted into the right femoral artery with a solid-state thermistor 5 mm from its tip, an injection device that connects to the distal lumen of a standard central venous catheter (inserted into the left internal jugular vein) and the PiCCO2 monitor (connected to the right femoral catheter). Finally, an epicardial echocardiography was performed using the Vivid S5 system (GE Healthcare, Germany) equipped with a 4 MHz probe (3Sc-RS, GE).

The established surgical protocol was applied (Morillas-Sendín et al., 2015; Quintana-Villamandos et al., 2021). A Biomedicus 540 centrifugal pump was implanted in the mini-pigs undergoing continuous-flow support. After median sternotomy, the animal was heparinised (4 mg/kg), a partial aortic cross-clamp was applied (just for anastomosing the output cannula of the LVAD to the aorta), and at 2 cm an aortotomy was performed. The output cannula of the LVAD was anastomosed to the ascending aorta, and the input cannula was placed through the apex of the left ventricle. Finally, both cannulas were connected to the device. Input flow was measured using an ultrasound transducer attached to the input cannula of the device.

### Experimental protocol and measurements

**FIGURE 1:**
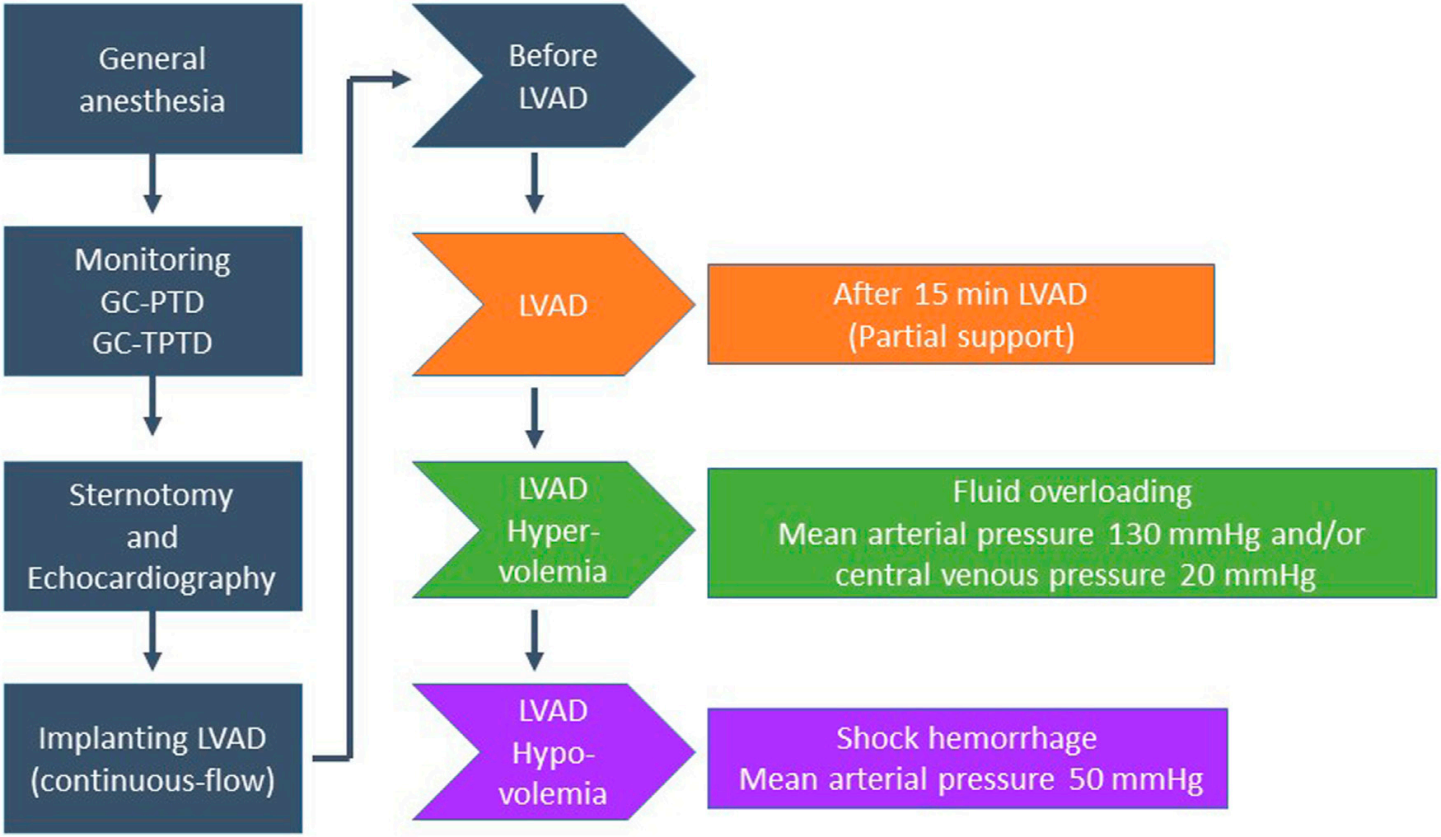
Study design (experimental procedure). Left ventricular assist devices (LVADs).

The experimental protocol is summarised in Figure 1. After sternotomy, an epicardial echocardiography was performed, and we excluded pigs with aortic insufficiency, tricuspid regurgitation, atrial septal defects or ventricular septal defects from the study. After implanting the LVAD, console parameters were adjusted to obtain a pump flow of 50% (partial support) of the baseline CO (before the LVAD is initiated) using the PAC. Measurements of CO using both methods—CO_PTD_ and CO_TPTD_—were obtained before starting the LVAD, and then with partial support LVAD during three periods (after 15 min of LVAD, after inducing hypervolemia, and after inducing hypovolemia). We induced hypervolemia with fluid overloading (75% serum saline + 25% gelatine) to increase the mean arterial pressure to 130 mmHg and/or the central venous pressure up to 20 mmHg, and we induced hypovolemia by controlled hemorrhage to decrease the mean arterial pressure to around 50 mmHg (Bendjelid et al., 2010).

### Data analysis

The results are expressed as mean + standard deviation (SD). CO determinations using the two methods, CO_PTD_ (standard technique) and COT_PTD_, were compared at different moments using paired Student’s *t*-test. Statistical significance was set at *p* < 0.05. Bias was defined as the mean difference between the two methods. A comparison of the bias between the two methods was assessed by Bland–Altman analysis (Odor et al., 2017). The percentage error was calculated as two SDs of the bias divided by the mean of the CO obtained by the standard technique. A percentage error of less than ±30% is recommended as clinically acceptable when comparing a new method to the current reference method (Odor et al., 2017). Linear regression analysis was used to assess the relationship between the CO values obtained by the two methods. The statistical analysis was performed using IBM SPSS Statistics for Windows, version 20.0 (IBM Corp, Armonk, NY, United States) and GraphPad Prism 6.0 (GraphPad Software, CA, United States).

## Results

In the present study, a mini-pig presented with atrial septal defects (detected by echocardiography) and was therefore excluded from the study (this is crucial to prevent end-organ damage due to hypoxemia by a right–left atrial shunt) (Quintana-Villamandos et al., 2012). A total of 72 paired measurements (three measurements from each stage per animal) were obtained from six consecutive mini-pigs. The reproducibility of the CO values over the study period is reported in Table 1.

**TABLE 1:** Cardiac output over the study period.

	Before LVAD	LVAD 15 min	LVAD Hypervolemia	LVAD Hypovolemia
CO_TPTD_ (L/min)	3.15 ± 0.72	3.27 ± 0.60	5.35 ± 0.57^*^	2.09 ± 0.57^*^
CO_PTD_ (L/min)	3.06 ± 0.64	3.23 ± 0.85	5.26 ± 0.71^*^	2.04 ± 0.53^*^

LVAD, left ventricle assist device; CO_TPTD_, cardiac output by transpulmonary thermodilution; CO_PTD_, cardiac output by pulmonary artery thermodilution. Results are expressed as the mean ± standard deviation. *p < 0.05 LVAD (15 min) vs. before LVAD or hypervolemia LVAD vs. LVAD (15 min) or LVAD hypovolemia vs. LVAD hypervolemia. **p < 0.05 CO_TPTD_, vs. CO_PTD._ *n* = 6 mini-pigs.

**FIGURE 2:**
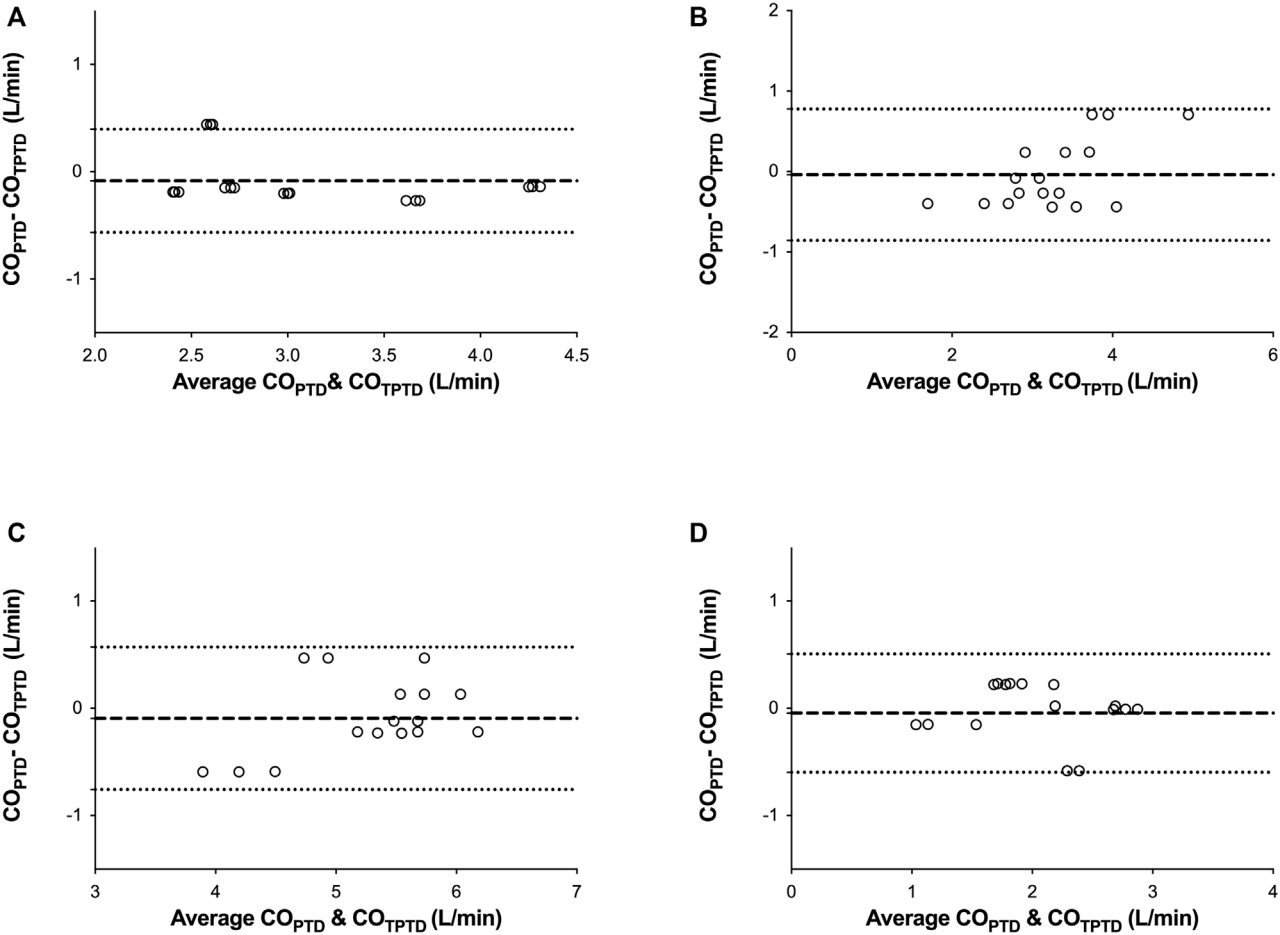
Correlation between the cardiac output (CO) measurements obtained by transpulmonary thermodilution (CO_TPTD_) and intermittent pulmonary artery thermodilution (CO_PTD_) in four consecutive moments of the study: before starting the left ventricular assist devices (LVADs) **(A)**, with the LVAD started in normovolemia **(B)**, hypervolemia (fluid overloading) **(C)**, and hypovolemia (shock hemorrhage) **(D)**.

**FIGURE 3:**
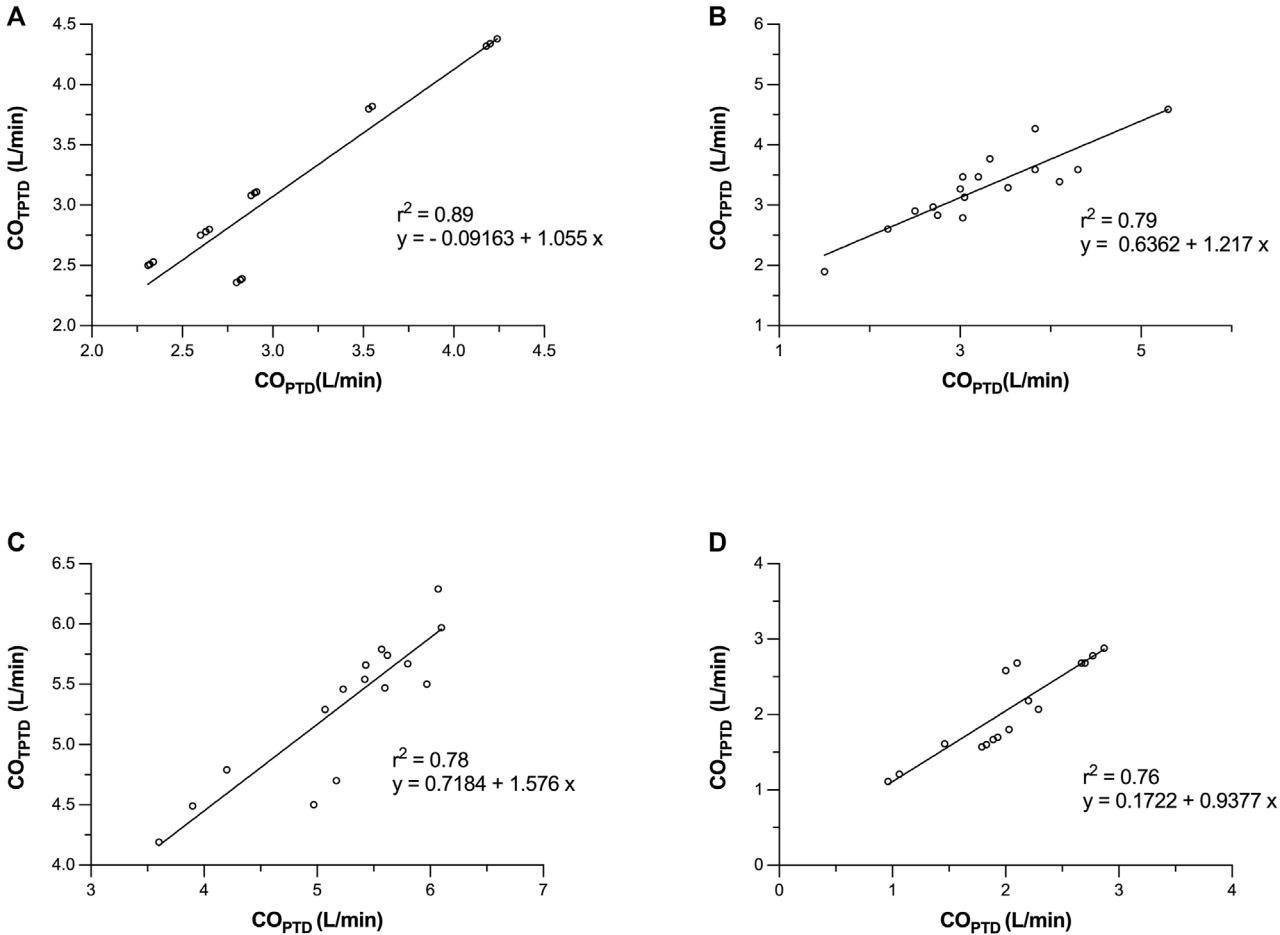
Bland–Altman representation depicting the agreement between the cardiac output (CO) measurements obtained by transpulmonary thermodilution (CO_TPTD_) and intermittent pulmonary artery thermodilution (CO_PTD_) in four consecutive moments of the study: before starting the left ventricular assist devices (LVADs) **(A)**, with the LVAD started in normovolemia **(B)**, hypervolemia (fluid overloading) **(C)**, and hypovolemia (shock hemorrhage) **(D)**.

Before starting LVAD, there were no significant differences between the measured CO_TPTD_ and CO_PTD_ values (3.15 ± 0.72 vs. 3.06 ± 0.64 L/min, respectively, *p* = 0.16). CO_TPTD_ and CO_PTD_ were closely correlated (*r* ^2^ = 0.89), with a mean bias (± SD) of −0.085 ± 0.245 L/min and percentage error of 16% (Figures 2A, 3A).

After 15 min of partial support, there were no significant differences between CO_TPTD_ and CO_PTD_ (3.27 ± 0.60 vs. 3.23 ± 0.85 L/min, respectively, *p* = 0.68). CO_TPTD_ and CO_PTD_ were closely correlated (*r* ^2^ = 0.79), with a mean bias (± SD) of −0.040 ± 0.417 L/min and a percentage error of 26% (Figures 2B, 3B).

After hypervolemia was induced, there were no significant differences between CO_TPTD_ and CO_PTD_ (5.35 ± 0.57 vs. 5.26 ± 0.71 L/min, respectively, *p* = 0.26). CO_TPTD_ and CO^PTD^ were closely correlated (*r* ^2^ = 0.78), with a mean bias (± SD) of −0.093 ± 0.339 L/min and a percentage error of 13% (Figures 2C, 3C).

After hypovolemia was induced, there were no significant differences between CO_TPTD_ and CO_PTD_ (2.09 ± 0.57 vs. 2.04 ± 0.53 L/min, respectively, *p* = 0.51). CO_TPTD_ and CO_PTD_ were closely correlated (*r* ^2^ = 0.76), with a mean bias (± SD) of −0.045 ± 0.281 L/min and a percentage error of 28% (Figures 2D, 3D).

## Discussion

The PiCCO system was compared to PAC in LVAD, and the results showed satisfactory agreement of CO values in a porcine model of normovolemia, hypervolemia and hypovolemia.

PAC is the clinical reference standard for CO measurement in critically ill patients (Vincent, 2012; Garan et al., 2020). PAC is still recommended by the interdisciplinary S3 guidelines in a cardiac surgical setting, and this indication includes patients with LVADs (Kanchi, 2011; Habicher et al., 2018). Continuous thermodilution by PAC has been validated in both continuous-flow (Quintana-Villamandos et al., 2021) and pulsatile-flow (Mets et al., 2002) LVADs, because it is crucial to know the status of end-organ perfusion in these patients (Birati and Rame, 2014). In a previous study, our group has validated continuous thermodilution by PAC using intermittent thermodilution by PAC (standard technique), in six minipigs with continuous-flow LVADs (Quintana-Villamandos et al., 2021). The study demosntrated that continuous thermodilution by PAC could be an alternative method of measuring CO in a continuous-flow LVAD in a porcine model of normovolemia, fluid overload, and bleeding. However, thermodilution by PAC is an invasive technique that has been associated with several complications (i.e. arrhythmia, coiling, knotting, carotid puncture, pulmonary infarction, intrapulmonary bleeding, pulmonary embolism, infection, pericardial effusion, pulmonary artery rupture and cardiac valvular damage) (Bossert et al., 2006; Evans et al., 2009; Sangkum et al., 2016; Benito-Saz et al., 2019). Thus, in the present work, our group studies a new CO monitoring (the PiCCO system) in a continuous-flow LVAD in a porcine model of normovolemia, fluid overload, and bleeding. We used another group of six minipigs to validate the PiCCO system (transpulmonary thermodilution) using intermittent thermodilution by PAC (standard technique). This is the first study to show the PiCCO system as an alternative to PAC in the measurement of CO in a porcine model with a continuous-flow LVAD.

Technological advances are necessary for minimally invasive or noninvasive CO measurement. Alternatives to the PAC have been developed, including transoesophageal echocardiography, arterial wave contour analysis and transpulmonary thermodilution (De Backer and Vincent, 2018). The PiCCO device uses a combination of transpulmonary thermodilution and pulse contour analysis. It is comparable with PAC thermodilution (Chakravarthy et al., 2007; Monnet and Teboul, 2017). CO measurement using the PiCCO can be influenced by valve pathology (i.e. tricuspid regurgitation and aortic insufficiency), intracardiac shunts, extracorporeal circulations and changes in body temperature. These potential sources of error are common to both PiCCO and PAC (Heerdt et al., 2001; Sami et al., 2007).

The PiCCO system is the most widely used monitor that employs transpulmonary thermodilution to measure CO. Intermittent bolus transpulmonary thermodilution is based on the Stewart–Hamilton equation; a cold saline bolus is introduced into the circulation through a central venous catheter, in which the external temperature sensor is located. Once in circulation, the thermistor located at the tip of the arterial catheter detects variations in the temperature, generating a thermodilution curve (Litton and Morgan, 2012). Transpulmonary thermodilution by PiCCO shows advantages relative to pulmonary thermodilution by PAC. PiCCO is less invasive (while it requires a central venous catheter and an arterial catheter, these are already required for all critical patients), and therefore it has few associated complications (e.g. inflammation and catheter-related infection (Sangkum et al., 2016). It is independent of respiratory cycles and able to measure parameters other than CO, including the extravascular lung water (to measure subclinical pulmonary edema), the global end-diastolic volume (volume in the heart at end-diastole) and the intrathoracic blood volume (volume in the heart and pulmonary circulation), both of which are used to estimate the cardiac preload (Litton and Morgan, 2012).

CO_TPTD_ does not provide a continuous measurement and needs calibration after acute haemodynamic changes (Monnet and teboul, 2017; Rozental et al., 2021). However, the reproducibility of CO_TPTD_ is higher (7% precision) than that of CO_PTD_ (15% precision) (Giraud el al., 2017; Rozental et al., 2021). The good reproducibility of transpulmonary thermodilution is related to the longer transit time of the thermal bolus (20 s), which reduces the artefacts produced by respiration, compared to pulmonary thermodilution (3–4 s). PAC may offer some advantage in patients with right heart dysfunction (De Backer and Vincent, 2018). However, in the present study we use a porcine model with a continuous-flow LVAD without right heart failure. PAC provides a series of measurements that are important for the management of patients with cardiogenic shock, especially right heart failure or adult respiratory distress syndrome. In these cases, the measurement of right cardiac output or the evaluation of pulmonary haemodynamics (in order to diagnose the presence of pulmonary hypertension and guide treatment) remain an indispensable part of proper monitoring (Monnet and Teboul, 2017). Furthermore, it also allows to determine the filling pressures of the left ventricle by occluding the pulmonary artery with the distal balloon and measuring the pulmonary artery occluded pressure. This parameter represents the filling pressures of the left atrium, and its morphology could contribute a lot of information to diagnose, for example, a significant mitral regurgitation. An infrared detector at the end of the pulmonary artery catheter allows continuous measurement of mixed venous saturation. It offers a real-time determination of the oxygen supply - demand ratio with good accuracy in real time (Bootsma et al., 2022a; Bootsma et al., 2022b). Transpulmonary thermodilution (by PiCCO device) is an alternative to PAC as a measure of CO (Litton and Morgan, 2012; Beurton et al., 2019). However, the use of PiCCO in patients with LVAD requires further validation. In the present study, we show that transpulmonary thermodilution could be an alternative method of monitoring CO in an LVAD in a porcine model.

We recognise some limitations of this study that should be noted. First, in the present work we study the CO_TPTD_ by PiCCO in a porcine model with a continuous-flow LVAD. However, the pulsatile-flow and continuous-flow LVADs show differences in hemodynamic response and ventricular unloading, and thus it is necessary to investigate monitoring systems on both LVADs (Cheng et al., 2014). Second, the CO measurements obtained from PAC are influenced more by the respiratory cycle than the transpulmonary thermodilution; however, in the present study, all measurements were made at the end of expiration when the variations are minimal. Finally, the LVAD is designed to be used in patients with heart failure; however, it is possible to study the validity of transpulmonary thermodilution for the determination of CO in LVAD in a healthy porcine model because the native ventricle can adjust to LVAD unloading by reducing flow through the aortic valve. Further investigations are necessary to reproduce these results in patients with heart failure.

## Conclusion

In conclusion, this study shows a good agreement between transpulmonary thermodilution by PiCCO monitor and pulmonary thermodilution by PAC in the intermittent measurement of CO in a porcine model with a continuous-flow LVAD.

## Data Availability

The original contributions presented in the study are included in the article/Supplementary Material; further inquiries can be directed to the corresponding author.
